# Image-Guided Radiotherapy and -Brachytherapy for Cervical Cancer

**DOI:** 10.3389/fonc.2015.00064

**Published:** 2015-03-17

**Authors:** Suresh Dutta, Nam Phong Nguyen, Jacqueline Vock, Christine Kerr, Juan Godinez, Satya Bose, Siyoung Jang, Alexander Chi, Fabio Almeida, William Woods, Anand Desai, Rick David, Ulf Lennart Karlsson, Gabor Altdorfer, The International Geriatric Radiotherapy Group

**Affiliations:** ^1^Medicine and Radiation Oncology PA, San Antonio, TX, USA; ^2^Department of Radiation Oncology, Howard University, Washington, DC, USA; ^3^Department of Radiation Oncology, Lindenhofspital, Bern, Switzerland; ^4^Department of Radiation Oncology, Centre Val d’Aurelle, Montpellier, France; ^5^Florida Radiation Oncology Group, Department of Radiation Oncology, Jacksonville, FL, USA; ^6^Department of Radiation Oncology, University of Arizona, Tucson, AZ, USA; ^7^Department of Radiation Oncology, University of West Virginia, Morgantown, WV, USA; ^8^Southwest PET/CT Institute, Tucson, AZ, USA; ^9^Department of Radiation Oncology, Richard A. Henson Cancer Institute, Salisbury, MD, USA; ^10^Department of Radiation Oncology, Akron City Hospital, Akron, OH, USA; ^11^Department of Radiation Oncology, Michael D. Watchtel Cancer Center, Oshkosh, WI, USA; ^12^Department of Radiation Oncology, Marshfield Clinic, Marshfield, WI, USA; ^13^Department of Radiation Oncology, Camden Clark Cancer Center, Parkersburg, WV, USA

**Keywords:** cervical cancer, IGRT, IGBT, normal tissue sparing

## Abstract

Conventional radiotherapy for cervical cancer relies on clinical examination, 3-dimensional conformal radiotherapy (3D-CRT), and 2-dimensional intracavitary brachytherapy. Excellent local control and survival have been obtained for small early stage cervical cancer with definitive radiotherapy. For bulky and locally advanced disease, the addition of chemotherapy has improved the prognosis but toxicity remains significant. New imaging technology such as positron-emission tomography and magnetic resonance imaging has improved tumor delineation for radiotherapy planning. Image-guided radiotherapy (IGRT) may decrease treatment toxicity of whole pelvic radiation because of its potential for bone marrow, bowel, and bladder sparring. Tumor shrinkage during whole pelvic IGRT may optimize image-guided brachytherapy (IGBT), allowing for better local control and reduced toxicity for patients with cervical cancer. IGRT and IGBT should be integrated in future prospective studies for cervical cancer.

## Treatment of Cervical Cancer

Radiotherapy is an excellent modality for the treatment of cervical cancer because of the tolerance of the cervix to high-radiation dose. Traditionally, staging and treatment of cervical cancer are based on clinical examination. The conventional radiotherapy technique is 3-dimensional conformal radiotherapy (3D-CRT) of the pelvis followed by 2-dimensional (2D) intracavitary brachytherapy. Tumor shrinkage during whole pelvic radiation allows for a better geometric implant leading to excellent local control and survival in patients with early stage disease. However, for patients with bulky disease or with locally advanced stages, loco-regional recurrences remain significant leading to a poor survival. The combination of chemotherapy and radiation has improved the prognosis of these patients but toxicity of the combined modality remains significant (1, 2). Grade 3–4 hematologic toxicity, radiation enteritis, and cystitis are often the limiting factors of the conventional radiotherapy technique and may compromise treatment efficacy because of treatment breaks, which allow tumor regrowth. Thus, a radiotherapy technique that spares the normal pelvic organs from excessive toxicity may reduce the acute side-effects and late complications of radiotherapy and potentially improve local control by an improved geometry of the brachytherapy implant. Image-guided radiotherapy (IGRT) based on modern imaging technology such as [^18^F]fluorodeoxyglucose positron-emission tomography (FDG-PET) scan and magnetic resonance imaging (MRI) may improve the therapeutic ratio and potentially decrease treatment toxicity.

## The Role of Pet Scan in Radiotherapy Planning for Cervical Cancer

Lymph node metastasis is one of the poor prognostic factors for cervical cancer. Compared to computed tomography (CT) scan and MRI, FDG-PET is more sensitive for detecting pelvic and para-aortic lymph node metastasis (3, 4). Among 560 patients with cervical cancer stage IA–IVB who underwent FDG-PET for staging at diagnosis, 47% had lymph node metastasis (5). Among the patients with PET-positive lymph nodes, all had pelvic, 35% para-aortic, and 12% supraclavicular lymph node metastasis. Thus, PET, by virtue of its lymph node detection, can upstage the clinical stage, modify treatment decision making, and allow the radiation oncologist to extend the radiotherapy volume for inclusion of the metastatic lymph nodes. The para-aortic lymph nodes can be treated with intensity-modulated radiotherapy (IMRT) achieving excellent regional control and acceptable morbidity (6). The feasibility of PET scan for para-aortic lymph nodes detection and radiotherapy planning was tested in a randomized trial. One hundred twenty-nine cervical cancer patients stage I–IVA with positive pelvic and negative para-aortic lymph nodes on staging MRI were randomized to have FDG-PET (*n* = 66) or no additional PET for staging (*n* = 63). Among patients who had para-aortic lymph nodes metastasis on PET scan, the radiotherapy fields were extended to include these metastatic lymph nodes. Seven patients had extra-pelvic metastases on PET scan: six of them para-aortic and one omental metastases. Even though there was no difference in survival between these two groups of patients, the ones who were randomized to PET scan had decreased para-aortic recurrences (7). As with all diagnostic modality, false negative results occur with PET staging. In a study of 237 patients with cervical cancer and negative para-aortic involvement on PET scan, 29 patients (12%) had occult para-aortic metastases on laparoscopic lymphadenectomy (8). Radiotherapy fields were extended to include these lymph nodes. However, among the 29 patients who had occult metastases, poor survival was observed in 16 patients who had para-aortic lymph nodes more than 5 mm in size raising the question about the benefit of lymphadenectomy in patients who had negative para-aortic lymph nodes on PET scan given the cost and the morbidity of the surgical procedure. FDG-PET can also be integrated in the IGRT treatment planning to escalate radiation dose to the positive lymph nodes with the simultaneous integrated boost (SIB) technique, potentially improving regional control (9). Even though patients with pelvic and or/para-aortic lymph nodes metastases often developed distant metastases, regional control with increased radiation dose to the metastatic lymph nodes may improve patient quality of life (10). Thus, despite its limitations, FDG-PET should be included in all IGRT planning for cervical cancer to assess the risk of lymph node and distant metastasis.

## The Role of MRI for Radiotherapy Planning of Cervical Cancer

Traditionally, staging and radiotherapy planning of cervical cancers are obtained through clinical information. However, clinical examination alone is often inaccurate to assess the local extension of the tumor especially its size, parametrium involvement, and pelvic side wall invasion. Even though MRI is less sensitive than FDG-PET for the detection of lymph node metastasis, its accuracy in the diagnosis of soft tissue tumor invasion and for the monitoring of tumor regression during radiotherapy makes this diagnostic imaging study indispensable for radiotherapy planning (11). Compared to CT scan, the T2-weighted images on MRI provide better resolution to outline the primary tumor and adjacent soft tissue invasion (parametrium, bladder, and rectum) due to its high soft tissue contrast (12). Following whole pelvic radiation, MRI is more accurate for the delineation of the residual gross tumor compared to both CT scan and clinical examination under anesthesia (13). The superiority of MRI in detecting residual disease following external beam irradiation for adaptive brachytherapy planning was also corroborated in another study (14). Serial MRI during whole pelvic radiotherapy may also predict the probability of local recurrence and poor survival. In a study of 80 cervical cancer patients stage IB2–IVA undergoing concurrent chemoradiation, the tumor volume was measured with MRI before (V1), at 2–2.5 weeks (V2), at 4–5 weeks (V3), and following treatment (V4). Large tumor size and poor tumor regression during chemoradiation were predictors of poor prognosis. Patients with a tumor volume >40 cc before treatment (V1) and a tumor ratio V3/V1 of 20% or more at the fourth or fifth week of whole pelvic radiation had a local recurrence rate of 63% and a disease-free survival of 39% at 5 years (15). A correlation between tumor regression during pelvic radiotherapy and survival was also observed in another study of cervical cancer (16). Even though these data are preliminary and need to be corroborated by further prospective trials, cervical cancer patients with large tumor and poor tumor regression during pelvic radiotherapy may be candidates for radiation dose escalation with brachytherapy. MRI-based brachytherapy planning allows for higher tumor dose and sparing of radiosensitive organs such as the rectum and bladder compared to conventional 2-D planning (17). The potential of MRI-guided planning optimization in intracavitary radiotherapy to increase tumor dose without excessive irradiation of the normal pelvic organs was also corroborated in another study (18). Increasing tumor dose to large tumors with MRI-based image-guided brachytherapy (IGBT) may improve local control and needs to be investigated in future prospective studies (14). As most radiation oncologists lack training in diagnostic radiology, perhaps the most challenging aspect of MRI-based target volume delineation is the uncertainty in outlining the target volume (19). As a result, radiation dose to the target and normal organs at risk (OAR) for complications may differ depending on individual delineation of the target volume (20). Inclusion of an experienced diagnostic radiologist specialized in gynecologic malignancies in the treatment team may improve this issue. Recently, functional MRI, such as diffusion-weighted imaging (DWI), has been investigated as a non-invasive biomarker for tumors. As the tumor shrinks with treatment, water mobility increases. Thus, the apparent diffusion coefficient (ADC) may increase and may serve as an indicator of tumor response. Preliminary studies using DWI–MRI as an early biomarker to assess tumor response following concurrent chemoradiotherapy have been promising, raising the need for future prospective studies (21).

## Potential Role of IGRT in Cervical Cancer

Intensity-modulated radiotherapy has been introduced to reduce treatment toxicity of whole pelvic irradiation compared to 3D-CRT. The steep dose fall-off of IMRT decreases significantly radiation dose to the normal pelvic organs. Grade 3–4 hematologic toxicity was significantly reduced in patients with cervical cancer undergoing weekly cisplatin and IMRT (22). Gastro-intestinal toxicity was also decreased with IMRT even though a large volume of the bowels was irradiated in patients with cervical cancer and para-aortic lymph node metastase (23). Excellent loco-regional control was also observed with acceptable toxicity in patients receiving postoperative IMRT and chemotherapy for high-recurrence risk features (lymph node metastases, positive margins, and parametrial invasion) (24). Thus, IGRT by combining the steep dose gradient of IMRT and daily imaging may further decrease the toxicity of whole pelvic irradiation in patients with cervical cancer because the planning target volume (PTV) may be safely reduced without any compromise on target coverage. Preliminary data suggest a dosimetric advantage of IGRT over IMRT for normal organ sparing in patients with cervical cancer. The dosimetric plans of 20 patients with cervical cancer stage IB–IVA undergoing IGRT and chemotherapy were retrospectively compared with IMRT. Even though both techniques provided optimal target coverage, IGRT significantly decreased radiation dose to the bowels compared to IMRT (25). The superior bowel sparing of IGRT over IMRT was also corroborated in another study of locally advanced cervical cancer (26). The bladder dose was also significantly reduced when volumetric arc therapy (VMAT) was compared to fixed beam IMRT (27). As VMAT is currently integrated into image-guidance radiotherapy for treatment delivery, VMAT-based IGRT may further improve normal organ sparing. The dosimetric advantages of IGRT were translated into low treatment morbidity in preliminary clinical studies of cervical cancer with this new technique of radiation. Among 15 patients undergoing chemoradiation for stage IB–IVA with PET-based IGRT, only 1 patient developed long-term chronic gastro-intestinal toxicity even though 4 patients received para-aortic lymph node irradiation (28). Another study corroborated the safety and efficacy of IGRT for locally advanced cervical cancer (29). As cervical cancer patients with a large tumor size at diagnosis are at high risk of local recurrence following radiotherapy, IGRT may deliver a higher dose to the gross tumor and areas at high risk for recurrence with the SIB technique without increasing radiation dose to the adjacent normal organs. The feasibility of IGRT to increase radiation dose to regions of resected metastatic lymph nodes was reported in 20 patients with stage IBpN1–IIIB cervical cancer undergoing primary chemoradiation after pelvic and para-aortic lymphadenectomy. The gross tumor, regional lymph nodes, and parametrium were treated to 50.4 Gy in 1.8 Gy/fraction whereas the regions of metastatic lymph nodes were treated to 59.36 in 2.12 Gy/fraction. Grade 3 diarrhea and neutropenia occurred in 5 and 25% of the patients, respectively, during whole pelvic IGRT. All patients underwent high dose rate (HDR)-based IGBT following pelvic IGRT (30). Thus, radiation dose escalation may be safe when IGRT is integrated with IGBT. An update of the study with 40 patients did not report any increase of grade 3–4 toxicity. In addition, complete pathologic response was confirmed by curettage 3 months following chemoradiation in 38/40 patients (31). As tumor regression carries a good prognosis, this investigative study is promising but needs to be confirmed by future prospective studies. The feasibility of IGRT for gross tumor dose escalation was also reported in another study of six patients with stage IB–IIB cervical cancer. The GTV and grossly enlarged lymph nodes and the parametrium, upper third of the vagina and the pelvic lymph nodes were treated to 59.8 Gy in 2.1 Gy/fraction and 50.4 Gy in 1.8 Gy fraction, respectively. Significant regression of the GTV was observed without increased radiation dose to the normal OAR for complications (32). In patients who are not suitable for intracavitary implants following pelvic irradiation because of poor geometry or co-morbidity, IGRT may deliver a high-boost dose to the gross residual tumor without significant treatment toxicity and improve local control (33). Another study corroborated the feasibility of IGRT boost for cervical cancer patients unable to undergo intracavitary implant (34). Even though these studies are preliminary, they suggest that IGRT by virtue of its steep dose gradient may produce a radiation dose distribution similar to the one performed with brachytherapy and allow a boost dose that can spare the OAR (35).

## Potential Role of IGBT in Cervical Cancer

Conventional intracavitary brachytherapy for cervical cancer relies on point dose and 2-D treatment planning based on conventional radiography without conforming to the tumor shape and size. Point A is often the reference point for radiotherapy dose delivery and the lack of dose-volume histogram (DVH) of the target volume and normal OAR make estimation of complications risks following radiotherapy difficult. The definition of point A also varies depending on the institution making radiation dose comparison between different radiation centers problematic. The lack of tumor visualization may lead to under-dosing of the tumor and over-dosing of the adjacent normal organs and may result in tumor recurrences and late complications. The introduction of advanced imaging into treatment planning allows for clear visualization of the tumor and the normal OAR, which may translate into better local control and survival and potentially less complications. MRI-based brachytherapy remains the gold standard for IGBT because of its high-soft tissue resolution allowing accurate delineation of the gross tumor and possible tumor invasion of adjacent normal organs. Standardization of target and OAR delineation and radiation dose delivery according to international organizations such as the guidelines of the Groupe Europeen de Curietherapie/European Society for Therapeutic Radiology and Oncology (GEC-ESTRO) may allow for compilation of DVH data between various institutions and ultimately establish a relationship between dose delivery, local control, and complications risks (36). As an illustration, the following targets were defined by GEC-ESTRO: GTV, high-risk clinical target volume (HR-CTV), and intermediate-risk clinical target volume (IR-CTV). Minimal dose delivered to 90% (D90) and 100% (D100) of the volume of interest should also be reported. By following GEC-ESTRO guidelines, many centers have achieved a satisfactory dose to the HR-CTV by using different planning methods (37). Adaptive planning in case of tumor regression between sequential brachytherapy sessions may further decrease the risk of complications because of a decreases radiation dose to the normal organs adjacent to the tumor (38). Thus, IGBT relies on solid scientific concepts allowing optimization of brachytherapy planning based on the tumor extent and the individual patient anatomy. Preliminary results suggest that compared to historic controls, IGBT may indeed improve local control and decrease late complications. Among 141 cervical cancer patients stage IB–IVA who had MRI-based IGRT according to GEC-ESTRO guidelines, local control was achieved in 134 patients (95%) at a median follow-up of 51 months (14). Local recurrences occurred in 35% of patients with a large tumor at diagnosis (>5 cm) and at the time of the implant (>5 cm). Regression of the tumor was a good prognostic factor as patients with large tumor at diagnosis and significant regression (<5 cm) during pelvic radiotherapy had a recurrence rate of 10.9%. There was a correlation between local control and the tumor dose for patients with large tumors. Local recurrence rate was 4 and 20% for HR-CTV D90 more than 87 and <87 Gy, respectively. An update of the study demonstrated a relationship between the dose to the rectum and late toxicities. Normal OAR DVH illustrates the dose to 2 cc (D2cc), 1 cc (D1cc), and 0.1 cc (D0.1cc) of the bladder and rectum from both external beam and brachytherapy. Grade 2–4 rectal side effects occurred in 5, 10, and 20% of patients for rectal D2cc of 67, 78, and 90 Gy, respectively (39). There was no significant correlation between bladder dose and late toxicities. This study suggests that IGRT may be complementary to IGBT because of the higher dose to GTV and lower dose to the rectum that can be achieved with IGRT compared to 3D-CRT. In another multi-centric study of 235 patients stage IIB–IIIB treated with pelvic chemoradiation followed by either 2D- (118) or 3D- (117) intracavitary brachytherapy, the local control rate was 73.9 and 78.5%, and grade 3–4 toxicity occurred in 22.7 and 2.6% of patients using 2D- and 3-D implant, respectively (40). Other studies also corroborated the high rate of local control achieved with IGBT with acceptable morbidity (41, 42). As an illustration, when MRI-based IGBT was retrospectively compared to CT-based external beam therapy and 2D-based brachytherapy, overall survival was significantly improved while severe late complications were reduced with IGBT (43). Thus, IGBT for cervical cancer can be performed in multiple institutions following standard guidelines with less complications compared to conventional 2-D and 3-D implants. The efficacy of IGBT for the treatment of cervical cancer has led some institutions to abandon hysterectomy in favor of definitive radiotherapy with IGBT in patients who traditionally required preoperative irradiation because of the tumor size (44). However, more prospective studies should be performed in the future to establish a clear relationship between tumor dose and local control, OAR DVH and late toxicity to establish IGBT as the standard of care for intracavitary implants.

The limitations of IGBT include the utilization of resources, which may be labor intensive and increases the financial burden of institutions with limited revenue. The use of an MRI for each individual brachytherapy fraction adds significantly to the treatment cost and may prevent IGBT implementation in many centers. A compromise would be to use MRI for the first fraction and CT-based plans for subsequent fractions. The feasibility of this approach was tested in a dosimetry study. Following the first MRI-based IGBT, the target structures delineated on MRI were loaded into the CT dataset while the OAR was contoured on the CT images (45). For small tumors, both MRI-based and hybrid-based plans were similar in terms of target coverage and OAR-constraints. Such innovative approach is intriguing and merits further investigation. Another limitation for the implementation of IGRT and IGBT in patients with cervical cancer is the shift of the normal organs during radiotherapy secondary to tumor regression and/or the filling of the bladder and rectum, which may result into higher dose to the OAR. Adaptive therapy is currently being investigated and may further improve the sparing of normal organs in the future (46).

## Conclusion

Image-guided radiotherapy and IGBT are promising radiotherapy techniques that can improve local control and decrease complication rates in patients with cervical carcinoma. The two image-based irradiation modalities are complementary and should be integrated in future prospective trials to improve patient quality of life and survival.

## Conflict of Interest Statement

The authors declare that the research was conducted in the absence of any commercial or financial relationships that could be construed as a potential conflict of interest.
